# The Role of Proteoglycans in Contributing to Placental Thrombosis and Fetal Growth Restriction

**DOI:** 10.1155/2011/928381

**Published:** 2011-03-03

**Authors:** Joanne M. Said

**Affiliations:** ^1^Level 7 Research Precinct, The Royal Women's Hospital, Locked Bag 300, Parkville, VIC 3052, Australia; ^2^Department of Obstetrics and Gynaecology, The University of Melbourne, Parkville 3052, Australia

## Abstract

Fetal growth restriction is an important pregnancy complication that has major consequences for the fetus and neonate as well as an increased risk of long-term morbidity extending into adulthood. The precise aetiology of most cases of fetal growth restriction is unknown although placental thrombosis is a common feature in many of these cases. This paper will outline the potential role of proteoglycans in contributing to placental thrombosis and fetal growth restriction.

## 1. Introduction

Adverse pregnancy outcomes remain a major cause of perinatal and paediatric morbidity and mortality, and subsequent morbidity into adult life. One of the key causes of adverse pregnancy outcome is fetal growth restriction (FGR). Fetal growth restriction describes a situation in which the fetus fails to achieve its full growth potential *in utero*. While an aetiology can be ascribed in a number of cases, the precise aetiology remains uncertain in up to 70% of cases. The ability to predict “at risk” pregnancies is limited, and therapeutic strategies are largely restricted to ultrasound surveillance for evidence of fetal decompensation secondary to hypoxia and then timely intervention by delivery. There is a trade off between delivering too early with the resultant risks of prematurity and delaying delivery with the attendant risks associated with hypoxia, neurological damage, and fetal demise. Of further concern is the accumulating evidence that growth-restricted fetuses are at increased risk of long-term postnatal sequelae including obesity, type II diabetes, hypertension, cardiovascular disease, and premature death [1]. While a number of maternal and fetal factors have been identified which contribute to FGR, the aetiologies of the vast majority of cases remain uncertain [2]. The pathogenesis of FGR appears to be related to abnormalities in the placental vascular flow [3] possibly due to microvascular thrombosis within the placenta [4]. The importance of understanding the mechanisms that control coagulation within the placenta cannot be overemphasised. Dysregulation of coagulation within the placenta is one of the few mechanisms which may potentially be amenable to therapy via anticoagulant treatment (including the development of specific targeted anticoagulants), hence it is imperative that this hitherto unexplored area of placental function be rigorously investigated.

## 2. Regulation of Thrombin within the Placenta

Normal human pregnancy is characterised by increased thrombin generation resulting in a shift towards a hypercoaguable state [5]. Amongst factors responsible for this alteration in systemic haemostasis is the possibility that excessive thrombin production may originate in the placenta [6]. Despite this, the placentae of normal human pregnancies are not characterized by excessive intravascular thrombosis, suggesting that factors within the placenta itself may offset the trend toward hypercoaguability within this organ [6]. In contrast, excessive uteroplacental thrombosis has been proposed as the pathogenic mechanism leading to a range of pregnancy complications including fetal growth restriction [4, 7, 8]. Histopathologic assessment of placentae from a range of pregnancy complications has demonstrated the presence of mural thrombi within both the maternal and fetal compartments of the placenta [9–12]. 

It is likely that the coagulation disturbance seen with FGR arises within the placenta itself rather than within the systemic circulation of either the fetus or the mother. Despite early work suggesting associations between inherited thrombophilias and adverse pregnancy outcomes [13, 14], numerous case-control studies [15, 16] and prospective cohort studies [17–19] examining maternal thrombophilias have failed to confirm significant associations between FGR and thrombophilias. Furthermore, recent prospective cohort studies have suggested that most patients with thrombophilias *do not *develop pregnancy complications and that most patients who do develop these complications *do not* carry a thrombophilic marker [17–19]. Likewise, several studies investigating the possible contribution of fetal or neonatal inherited thrombophilias to adverse pregnancy outcomes have failed to confirm this association [15, 16]. While it remains possible that the placental thrombosis that is observed in patients with these adverse pregnancy outcomes may be due to other (as yet unidentified) thrombophilic markers, a more likely explanation is that the coagulation disturbance arises within the placenta itself.

## 3. Proteoglycans

Proteoglycans are macromolecules located within vessel walls that contain a core protein to which sulphated glycosaminoglycan (GAG) chains are covalently linked. There are four types of GAG chains located in the blood vessel wall: chondroitin sulphate, dermatan sulphate, heparan sulphate, and hyaluronan [20]. However, only chondroitin sulphate, dermatan sulphate, and heparin sulphate are covalently linked to a core protein [20].

Placentae contain two major types of proteoglycans; those containing heparan sulphate and those containing chondroitin sulphate or dermatan sulphate [21]. Heparan sulphate proteoglycans in the placenta include syndecan and perlecan [22, 23] while chondroitin sulphate and dermatan sulphate containing proteoglycans in the placenta include decorin and biglycan [24]. Decorin usually has one chondroitin sulphate or dermatan sulphate GAG attached to its core protein, while biglycan normally has two of these GAGs attached to its core.

Swan et al. have previously demonstrated that decorin is localised to the stroma surrounding fetal blood vessels of placental villi [25], whereas Murthi et al. demonstrated that biglycan is localized to endothelial and smooth muscle cells of fetal capillaries [26]. In contrast, syndecan 1 was highly expressed in the syncytiotrophoblast [27].

## 4. Actions of Proteoglycans and GAGs in the Human Placenta

Proteoglycans and their GAG sidechains have been shown to have important actions within the placenta. These molecules display important anticoagulant actions which suggest they may play a key role in preventing thrombotic events within the placenta [6, 28]. Heparan sulphate chains bind Antithrombin through a pentasaccharide sequence, while dermatan sulphate chains bind heparin cofactor II (HCII) through a highly charged sequence [28] GAG bound antithrombin and HCII are thought to undergo a conformational change, which in turn facilitates the inhibition of thrombin [29, 30]. The cellular localization of PGs to endothelium and cells in contact with circulating blood, suggest an important role for these molecules in localized anticoagulation.

## 5. Could Proteoglycans Contribute to FGR?

Swan et al. demonstrated a significant reduction in the mRNA and protein expression of decorin in placentae from human pregnancies complicated by FGR compared to gestation matched controls [25]. Likewise, Murthi et al. demonstrated a significant reduction in the mRNA and protein expression of biglycan in FGR compared to gestation matched controls. Both studies confirmed the localization of these molecules in close proximity to circulating blood, supporting the hypothesis that localized anticoagulant activity may be reduced in FGR placentae. Whether these changes correlate with the increased placental thrombosis observed in FGR placentae remains uncertain. Likewise, it is plausible that the pathophysiological process resulting in FGR is not purely restricted to alterations in thrombin regulation, since decorin has been shown to interact with insulin-like growth factor-1 [31], thereby raising the possibility that altered decorin expression may interfere with growth factor expression and hence fetal growth. Decorin has also been shown to inhibit trophoblast proliferation and migration [32, 33] raising yet another potential mechanism by which altered decorin expression may play a role in the pathogenesis of fetal growth restriction. 

Fetal growth restriction is commonly associated with pre-eclampsia and hypertensive disorders of pregnancy [2]. Altered GAG sulphation patterns have been observed in placentae from pregnancies complicated by pre-eclampsia [34], raising the possibility that this could result in altered GAG function and thereby contribute to the development of pre-eclampsia. Chen et al. have also demonstrated upregulation of proteoglycan expression and alterations in the GAG composition in placentae from women with gestational diabetes and hyperglycaemia [24]. Gestational diabetes is typically associated with fetal overgrowth; however, increased fetal morbidity and mortality are well recognized [35]. It is plausible that altered placental proteoglycan homeostasis may be an important mechanism contributing to the poorer perinatal outcomes observed in all these pregnancy complications.

However, determining causation is not always easy in human pregnancy research so it remains uncertain whether the alterations in proteoglycan expression observed are truly causative, or rather reflect a response to an altered growth process. Ishiguro et al. has demonstrated alterations in placental vasculature in Syndecan 4 knockout mice with greater evidence of fibrin and calcium deposition suggesting disruption of the local anticoagulant mechanisms, although a statistically significant growth disturbance was not seen [36].

## 6. Potential Therapies

At present, there are no proven treatments for FGR. Understanding the potential role of proteoglycans in contributing to FGR is important as it may allow the rational use of therapeutic glycosaminoglycans (i.e., heparins) to augment the anticoagulant activities of proteoglycans. The challenge, however, is to ensure that this anticoagulant activity is delivered to the compartment of the placenta in which the functional defect can be demonstrated and that undesired effects of systemic anticoagulation for either mother or fetus can be avoided.

## 7. Conclusion

Fetal growth restriction is a serious pregnancy complication that cannot currently be “treated” during pregnancy. Management relies on appropriate detection and timely intervention by delivery to prevent the serious consequences of this condition. This often necessitates the delivery of extremely premature infants who require the investment of a considerable range of financial, intellectual, and emotional resources to ensure their survival. Furthermore, these infants are at risk of developing the long-term consequences of these conditions which may not become apparent until adulthood such as an increased risk of obesity, cardiovascular disease, and diabetes. Understanding the pathophysiological role of proteoglycans in the placenta may provide an important therapeutic window to prevent or treat fetal growth restriction in the future. There is no doubt that efforts to improve the fetal and neonatal outcomes for these infants appear well justified given the possible life-long improvements which may ensue.
